# A Review of the Effects of Physical Activity and Exercise on Cognitive and Brain Functions in Older Adults

**DOI:** 10.1155/2013/657508

**Published:** 2013-09-11

**Authors:** Louis Bherer, Kirk I. Erickson, Teresa Liu-Ambrose

**Affiliations:** ^1^PERFORM Centre, Concordia University, Montreal, QC, Canada; ^2^Research Center, Institut Universitaire de Gériatrie de Montréal, Montreal, QC, Canada; ^3^Department of Psychology, University of Pittsburgh, Pittsburgh, PA, USA; ^4^Department of Physical Therapy, University of British Columbia, Vancouver, BC, Canada; ^5^Brain Research Centre, University of British Columbia, Vancouver Coastal Health Research Institute, Vancouver, BC, Canada

## Abstract

Studies supporting the notion that physical activity and exercise can help alleviate the negative impact of age on the body and the mind abound. This literature review provides an overview of important findings in this fast growing research domain. Results from cross-sectional, longitudinal, and intervention studies with healthy older adults, frail patients, and persons suffering from mild cognitive impairment and dementia are reviewed and discussed. Together these finding suggest that physical exercise is a promising nonpharmaceutical intervention to prevent age-related cognitive decline and neurodegenerative diseases.

## 1. Introduction

Chronological aging, or senescence, is associated with an increased risk of chronic conditions and diseases such as cognitive impairment, cardiovascular disease, and metabolic syndrome. Due to prolonged life expectancy, age-related diseases have increased in alarming proportions in recent decades [1]. An increasing body of studies have suggested that lifestyle factors have a significant impact on how well people age. For example, Fratiglioni et al. [2] reported that three lifestyle factors can play a significant role in slowing the rate of cognitive decline and preventing dementia: a socially integrated network, cognitive leisure activity, and regular physical activity. In this review and others [3, 4], it is argued that out of these lifestyle factors, physical activity has the most support as protective against the deleterious effects of age on health and cognition. Broadly defined, physical activity refers to activity that is part of one's daily life involving bodily movements and the use of skeletal muscles. Physical exercise is a subcategory of physical activity that is planned, structured, and purposive to improve specific physical skills or physical fitness. Evidence suggests that physical activity and exercise can to some extent lower the risk of adverse outcomes associated with advancing age.

Physical activity maintained throughout life is associated with lower incidence and prevalence of chronic diseases such as cancer, diabetes and cardiovascular and coronary heart diseases [5, 6]. Recent studies suggest that physical exercise also protects against dementia [7]. Yet, despite this promise, the ways in which physical activity impacts the rate and prevalence of cognitive decline is still under investigation. Furthermore, several open issues call for further research, such as the dose-response relationship, the level of change or protection provided by physical activity, the biological and/or psychological mechanisms by which these effects occur and whether physical activity can be beneficial despite chronic medical conditions, neurological syndromes such as dementia, and the physical limitations observed in frail patients. Although recent advancements in neuroimaging techniques and genetics have opened new research avenues, more studies are required to provide definitive answers to these important questions. This literature review aims to provide an overview of studies that have attempted to assess whether and how physical activity and exercise positively impact older adults at any age and with various physical and psychological conditions. 

## 2. Aging and Neurocognitive Functions

It is generally assumed that age brings with it declines in performance in a multitude of cognitive tasks that require a variety of perceptual and cognitive processes (for extensive reviews of the literature see [3, 8, 9]). More specifically, processing speed declines early in the course of aging, which has recently been associated with loss of white matter integrity [10]. Working memory, or the ability to maintain and consciously manipulate information, is also highly age-sensitive. The age-related difference in working memory tends to be greater if executive control processes such as inhibition, updating, and manipulation are required, and even greater if the memory load (i.e., the number of items to be maintained) is high. These deficits have sometimes been associated with reduced task-related activation in older compared to younger adults in frontal regions of the cerebral cortex. Other studies also reported higher task-related activation in older adults, a phenomenon possibly associated with compensation for age-related changes in brain structure and functions [9]. Older adults also tend to show reduced inhibition compared to younger adults. As a result, they are more distracted by irrelevant information and more affected by proactive interference (i.e., interference induced by current learning on further encoding of new information). Furthermore, episodic memory declines in late adulthood, likely due to poor encoding strategies, less use of environmental support, and deficits in binding new information with existing knowledge during encoding.

Structural and functional brain imaging studies have provided insights into potential brain mechanisms of cognitive aging. For instance, changes in brain volume occur faster in adults after 50 years of age, with an annual decline of 0.35% compared to 0.12% in young adults (see [11, 12] for reviews). Ventricle dilatation can approximate 4.25% per year at 70 years of age compared to 0.43% in young adults. The volume of the hippocampus, a cerebral structure that plays a major role in memory formation, is also sensitive to age, with an annual decline of 0.86% per year (from 26 to 82 years), 1.18% per year after 50 years, and 1.85% per year after 70 years. Yet, rate of change is difficult to appreciate due to the lack of longitudinal studies. In a recent study, Raz et al. [13] followed participants over 30 months and observed that the hippocampus, the entorhinal cortex, the orbital-frontal cortex, and the cerebellum showed volumetric changes after only 15 months, while other brain structures showed shrinkage after 30 months, including the caudate nucleus, the prefrontal subcortical white matter, and the corpus callosum. However, some brain structures showed almost no change (the primary visual cortices, the putamen, and the pons). In addition, aging is associated with overall changes in white matter integrity (e.g., leukoaraiosis), with greater changes occurring after the seventh decade, and localized preferentially in the frontal and prefrontal regions [11]. These changes are more pronounced in patients with vascular diseases such as hypertension and type-2 diabetes. Cerebral metabolism is also altered by age, with a reduction in regional cerebral metabolic rate for glucose, oxygen, and blood flow. Although it is frequently assumed that structural changes are associated with a decline in brain metabolism, recent evidence suggests otherwise. For example, Chen et al. [14] observed that, in some brain regions, age-associated reductions in cerebral blood flow could occur independently of regional atrophy [14].

## 3. Physical Activity and Cognition in Healthy Seniors

Several studies support the notion that physical activity is a significant moderator of age-related cognitive decline. In cross-sectional studies, age-related differences in cognitive performance observed when older adults are compared to younger participants are reduced if the comparisons involved higher-fit individuals rather than sedentary older adults [15–19]. As a whole, these cross-sectional studies suggest that cardiorespiratory fitness is associated with more efficient cognitive functions.

In longitudinal studies, older adults that participate in physical activity show less cognitive decline over two- to 10-year follow-up periods. For instance, in a study by Barnes et al. [20] cardiorespiratory fitness assessed at baseline predicted cognitive performance six years later in a variety of cognitive domains (working memory, processing speed, attention, and general mental functioning). In nationally representative samples of noninstitutionalized persons aged 50 years and older and across 11 European countries (Austria, Germany, Sweden, Denmark, Switzerland, the Netherlands, Belgium, France, Spain, Italy, and Greece) Aichberger et al. [21] reported that individuals who participated in any type of regular physical activity showed less cognitive decline after 2.5 years, especially when they engaged in vigorous activities more than once a week. 

The impact of physical activity on cognition in older adults is more strongly supported by results from intervention studies, which generally show that older adults who have completed a physical activity program that produces significant increases in cardiorespiratory fitness (indexed by direct measures or estimation of VO_2max⁡_) often show enhanced cognitive performance. Dustman et al. [22] compared middle-aged and older individuals who completed a four-month aerobic training program to age-matched controls who participated in strength and flexibility exercises and controls who did not exercise. Only the aerobic training group showed improved cardiorespiratory function, along with improvements on a simple RT task. Similar results were obtained in women aged 57 to 85 years old following a three-year physical training program [23]. Hawkins et al. [24] reported that, in older adults, a 10-week aquatic fitness program led to greater improvement in task conditions that tap dual-task and switching abilities compared to conditions that do not require executive or attentional control processes. In Kramer et al.'s [25] study, older adults who completed a six-month aerobic training program (walking) showed a significant improvement in cognitive performance, unlike those who completed a stretching program. Cognitive improvement was greater in tasks that tapped attentional control or executive control functions and was correlated with improvement in VO_2max⁡_. In another study, Albinet et al. [26] reported that 12 weeks of aerobic training lead to enhanced performance in executive control and increased heart rate variability in older men and women aged 65–78. These results suggest that aerobic exercise may be an important cardiac and brain protective factor as people age. The greater improvement induced by aerobic training in executive control compared to other cognitive domains has also been confirmed by several meta-analyses (see [27] but see [28] for different conclusions).

The selective benefit of aerobic exercise for tasks that tap executive control was also observed in another recent study [29], where 57 older adults completed a 10-month training program (aerobic versus strength and flexibility). The positive effect on executive control was observed after aerobic training only. In another study, Renaud et al. [30] observed that only 12 weeks of aerobic training induced a significant improvement in cardiorespiratory capacity (estimated VO_2max⁡_) along with enhanced motor response preparation, such that participants maintained response preparation over time more efficiently after the training program. These results provide additional support for the notion that improving aerobic fitness may enhance attentional control mechanisms in older adults. In a meta-analytic review of randomized-control trials of aerobic exercise on neurocognitive functions, Smith et al. [31] examined 29 studies conducted between 1966 and 2009 (including more than 2,000 participants and 234 effect sizes). They found that individuals who were randomly assigned to aerobic exercise training showed modest improvements in attention, processing speed, executive function, and memory, with less convincing effects on working memory. These results, along with those from Colcombe and Kramer [27], suggest a selective effect of aerobic exercise on neurocognitive functions. However, not all studies reported a significant correlation between improvement in cardiorespiratory fitness outcomes and cognitive improvement (see [28, 32] meta-analysis), which suggests that physiological mechanisms supporting cognitive enhancement remain to be fully understood.

Studies reported above highlight that aerobic exercise enhances cognitive function. However, recent evidence now suggests that other types of exercise training, such as resistance training, may also benefit cognition. Cassilhas and colleagues [33] demonstrated that six months of either thrice-weekly moderate or high intensity resistance training improved memory performance and verbal concept formation among 62 community-dwelling senior men aged 65 to 75 years. Liu-Ambrose and colleagues [34] demonstrated that an individualized home-based program of balance and strength retraining significantly improved selective attention and conflict resolution as measured by the Stroop Test after six months among seniors aged 70 years and older with a recent history of falls. The finding of this study is notable given that many have hypothesized that the cognitive and neural benefits of exercise must occur within the context of social engagement for it to be effective [35]. Liu-Ambrose and colleagues [36] also demonstrated that 12 months of either once-weekly or twice-weekly progressive resistance training improved Stroop Test performance among 155 community-dwelling senior women aged 65 to 75 years. Enhanced selective attention and conflict resolution were also associated with increased gait speed. Clinically, improved gait speed predicts a substantial reduction in both morbidity [37] and mortality [38, 39]. These results illustrate the clinical significance of cognitive gains induced by resistance training. Therefore, it seems that in addition to endurance training, resistance training should be seriously considered as a potential modifier of cognitive functions in older adults. Recent studies also suggest that motor learning and coordinative exercise could also be used to enhance cognitive function in this population (see [40]). 

## 4. Physical Activity and Brain Structures and Functions in Older Adults 

The biological mechanisms by which cognition is enhanced through physical exercise training remain to be completely elucidated, although the number of studies that have tried to identify these mechanisms has increased in the last 10 years. For the most part, the studies that support the notion that physical exercise has an impact on brain functions have focused on direct biological effects of exercise using both animal and human models. However, as suggested by Spirduso et al. [41] exercise may enhance cognition indirectly by improving health conditions (stress, sleep) and reducing chronic diseases (coronary heart diseases) that impact neurocognitive functions. 

The evidence for the direct effects of exercise on the brain first came from animal studies. In a comprehensive literature review, Lista and Sorrentino [42] suggest that the basic neurobiological mechanisms associated with exercise can occur at two levels, supramolecular and molecular. At the supramolecular level, physical activity has been found to induce angiogenesis or the physiological process by which new blood vessels grow from preexisting vessels [43, 44]. Physical activity has also been associated with neurogenesis, or neural cell proliferation, in the hippocampus in elderly rats [45]. Although the functional significance of this effect remains unclear, there is evidence that newly formed neurons can integrate into a neural network and become functional [46]. Exercise-induced synaptogenesis has also been reported [47, 48]. 

The molecular mechanisms by which exercise induces angiogenesis, neurogenesis, and synaptogenesis have received growing attention in the last few years. Again, the evidence comes mainly from animal studies that showed exercise-associated changes in molecular growth factors such as brain-derived neurotrophic factor (BDNF), which plays a crucial role in neuroplasticity and neuroprotection, and increased production of insulin-like growth factor 1 (IGF-1), which is involved in both neurogenesis and angiogenesis. Moreover, neurotransmitter systems also seem to be modulated through exercise (see [42]). Until very recently, evidence for the molecular and supramolecular effects of exercise came exclusively from animal studies. However, a very innovative study [49] recently showed that greater exercise-related increases in BDNF were associated with increased hippocampal volume. If reproduced, these results would confirm that physical exercise induces genuine neurotrophic effects on brain structures and functions at the molecular, supramolecular, and structural levels. 

In humans, several studies using structural and functional brain imaging, or electrophysiological measures of brain activity, suggest that physical exercise induces transient and permanent changes at the structural and functional levels in the aging brain [50–54]. Using voxel-based morphometry (VBM), or detailed image segmentation of high-resolution brain scans, Colcombe et al. [55] reported that a higher cardiorespiratory fitness level (VO_2max⁡_) was associated with a reduced loss of grey and white matter in the frontal, prefrontal, and temporal regions in older adults. In another study, Erickson et al. [56] performed a region-of-interest analysis on magnetic resonance images in 165 nondemented older adults and found that higher fitness levels were associated with larger left and right hippocampi that further correlated with better spatial memory performance. These findings suggest that aerobic fitness is associated with changes in brain structures that translate into better cognitive function in older adults (see also [49, 57]). 

Even more striking evidence of the benefit of fitness on brain functions comes from functional brain imaging studies (fMRI). It has been shown that enhanced cardiovascular functions after aerobic training are associated with greater task-relevant activity in brain areas recruited in an attentional control task [58]. Similarly, 12 months of resistance training in community-dwelling senior women led to functional changes in two regions of the cortex previously associated with response inhibition processes, the anterior portion of the left middle temporal gyrus, and the left anterior insula extending into lateral orbital frontal cortex [36]. These hemodynamic effects cooccurred with improved task performance. Moreover, Voss et al. [59] found changes in functional connectivity after aerobic exercise training in older adults. They observed that 12 months of training leads to increased connectivity in regional connections that support both the default-mode network and the frontal executive network, suggesting that physical exercise has a restorative effect on large-scale brain circuitry. Changes in these large-scale brain networks have received increasing attention in aging neuroscience, as they indicate massive changes in brain systems.

A complete understanding of the potential for physical activity to protect the brain from the effects of age would require investigating the indirect influences of exercise on cognition. There is growing evidence that exercise has indirect beneficial effects on cognition through its impact on factors that are known to alter neurocognitive integrity [60]. Spirduso et al. [60] suggest three groups of potential mediators in the relationship between exercise and cognition: physical resources, chronic diseases or states, and mental resources. It has been shown that physical exercise enhances mental resources by reducing depression [61], anxiety, and chronic stress and improving self-efficacy [62]. The effect of physical activity on cognitive function might also be mediated by physical resources such as diet [63] and sleep [64, 65]. It remains to be seen whether these factors in fact mediate the positive effects that exercise has on cognitive and brain health.

## 5. Physical Activity and Cognition in Frail Older Adults 

With increasing age, and specifically with advanced age (i.e., over 75 years), many individuals eventually develop one or more of a group of related medical problems referred to as geriatric syndromes. Perceptual limitations (vision and hearing problems), urinary incontinence, falls, delirium, and dementia are examples of geriatric syndromes. These syndromes are characterized by having more than one cause and by involving several different body systems. An emerging symptom that appears particularly relevant to our purpose is frailty, as it apparently limits physical activity and exercise. Frailty is defined as a complex health state of increased vulnerability to stressors due to impairments in multiple systems. It has been associated with adverse outcomes such as disability, falls, hospitalization, and death [66]. With aging, the prevalence of frailty increases from 7% in older adults aged between 65 and 74 years to 18% between 75 and 84 years and 37% at age 85 years and older [67]. Physical inactivity is a major risk factor for frailty [66]. It is important to note that frailty is not a contraindication for physical activity. On the contrary, it may be one of the most compelling reasons to prescribe physical exercise [68].

Results from longitudinal studies show that physical activity and exercise can prevent frailty in older adults. In a recent study, 2,964 older adults were followed for five years to determine the relationship between physical activity and the risk of becoming frail [69]. Results showed that individuals who regularly exercised at baseline were less likely to develop frailty within a five-year period than sedentary individuals, even after adjusting for baseline health conditions and demographic characteristics. 

Intervention studies also suggest that physical activity can improve several frailty syndrome components, especially sarcopenia (reduction in skeletal muscle mass) and functional impairment [68]. Moreover, in a recent randomized controlled trial that assessed the impact of a three-month physical training intervention on quality of life in 77 physically frail persons aged 75 years and older [70], it was observed that functional exercises twice a day to improve balance and lower extremity muscle strength, in addition to strength training twice a week, helped to improve psychological well-being associated with physical functioning, emotion, and mental health. To our knowledge, only one study has shown that physical exercise training can help improve cognition in frail older adults. Langlois et al. [71] recently observed that three months of training in frail older adults resulted in significant improvement in both physical capacity and cognitive performance (executive functions, processing speed, and working memory) as well as quality of life associated with leisure activities and satisfaction with physical capacity.

## 6. Physical Activity and Cognition in Older Adults with Mild Cognitive Impairment and Dementia

According to the Alzheimer's Association [72], one in eight people aged 65 and older (13%) and 43% of people 85 and older have Alzheimer's disease. Currently, there is no cure for Alzheimer's disease. However, research has suggested that physical activity and exercise can significantly reduce the risk of developing it. In a recent cross-sectional study that compared 198 subjects with mild cognitive impairment (MCI) to 1,126 with normal cognition, Geda et al. [73] observed that moderate activity during midlife was associated with a 39% lower risk of having mild cognitive impairment in later life. Late-life moderate exercise was associated with a 32% lower risk for MCI. Burns et al. [74] explored the effect of exercise on cognitively impaired individuals and found an association between direct measures of cardiorespiratory fitness (VO_2_ peak) and cognition (neuropsychological test battery) in normal older participants and patients in the early stage of Alzheimer's dementia (AD). Results showed that cardiorespiratory fitness was modestly reduced in patients with AD compared to participants without dementia. Although no significant association was found between cardiorespiratory fitness and cognition in participants without dementia, higher fitness levels in early AD participants were associated with larger brain volume (less brain atrophy), even when controlling for age, sex, dementia severity, and physical frailty.

In a longitudinal study exploring the association between midlife physical activity and late-life cognitive function and dementia, Chang et al. [75] observed that being active (around 5 hours per week) was associated with higher scores in processing speed, memory, and executive functions, even after controlling for demographic and cardiovascular factors. Moreover, participants who reported being active were significantly less likely to have dementia in later life. In a prospective study following 1,740 persons older than 65 years without cognitive impairment for a period of 6.2 years, Larson et al. [7] reported reduced dementia incidence for individuals who exercised three or more times a week (13 per 1,000 person-years) compared to those who exercised fewer than three times a week (19.7 per 1,000 person-years), demonstrating a 32% reduced risk for dementia. 

Interestingly, correlations have also been reported between muscle strength and a lower risk of AD and a slower rate of cognitive decline. Having followed 900 community-based older persons without dementia at baseline, Boyle et al. [76] showed a lower rate of global cognitive decline, MCI and AD in older adults with higher muscle strength. The protective effects remained after adjustment for several covariates, including body mass index, physical activity, pulmonary function, vascular risk factors, vascular diseases, and apolipoprotein E4 status. Furthermore, in a recent meta-analysis of prospective studies that covered 15 prospective studies (12 cohorts) and 33,816 nondemented individuals, 3,210 of which showed cognitive decline during the one- to 12-year followup, Sofi et al. [77] observed that physical activity significantly and consistently prevented cognitive decline. Individuals who were highly physically active showed 38% less risk of cognitive decline, and those who did low-to-moderate level exercise also showed a significantly 35% reduced risk.

Results from intervention studies are scarce. A meta-analysis of randomized controlled trials (2,020 participants; 30 trials) [78] reported beneficial effects of physical activity on physical fitness (effect size = 0.69) and cognitive function (effect size = 0.57) in adults with cognitive impairment (MCI and dementia). However, other studies have reported modest benefits [79] or no appreciable effect [80] of physical activity on cognition in patients with cognitive impairment. A recent randomized clinical study evaluated the impact of a six-month aerobic exercise intervention in individuals with mild cognitive impairment [81]. Thirty-three older adults (17 women) with amnestic mild cognitive impairment ranging in age from 55 to 85 years were randomized to either a highintensity aerobic exercise (75–85% of heart rate capacity) or a stretching control group. Results showed beneficial effects of aerobic exercise, especially in speed of processing and executive functioning, although in some tests, gender differences in cognitive improvement were observed, despite comparable gains in cardiorespiratory fitness in men and women. In a randomized controlled study, Kemoun et al. [82] examined the benefits of a 15-week physical activity program in 31 subjects (mean age of 82 years). They reported that a physical activity program can slow cognitive decline and improve quality of walking in elderly persons with dementia. A more recent study assessed the cognitive impact of a Tai-Chi intervention group (*n* = 171) compared to a stretching and toning group (*n* = 218) in older adults with cognitive impairment [83]. Results showed that both groups improved in global cognitive function, delayed recall, and subjective cognitive complaints. However, improvements in balance, visual span, and Clinical Dementia Rating scores were observed in the intervention group only. Future studies are needed to specify whether interventions should involve aerobic or strength training exercises or both to improve cognition in MCI patients. Nagamatsu et al. [84] recently reported that patients who completed six months of aerobic or strength training exercise showed improved spatial memory performance, but only the aerobic group showed a correlation between physical capacity after intervention and spatial memory performance. The mechanisms by which exercise impacts cognition in this population thus deserve further study.

## 7. Conclusions

In recent decades, an increasing number of studies have suggested that people should adopt physical activity and exercise as part of their lifestyle to alleviate the negative impact of aging on the body and the mind. However, we still do not understand how physical activity impacts the rate of cognitive decline. One major issue is whether physical activity broadly defined (i.e., activity that is part of one's daily life involving bodily movements and the use of skeletal muscles) or structured exercise (i.e., physical activity that is planned, structured, and purposive to improve physical fitness) leads to the same benefits in preventing age-related cognitive decline. Physical exercise often differs from physical activity by being more controlled in terms of intensity and duration, while physical activity studies tend to incorporate a large variety of unspecified activities. Future studies are required to understand the intensity, duration, and types of exercise that better enhance cognitive functions in older adults. Although recent advancements in brain imaging techniques and genetics have opened new research avenues, more studies are required to find definitive answers to these questions. Further research is needed to better document the impact of other forms of exercise, as well as the dose-response relationship that governs the positive impact of exercise on brain functions. Hopefully, ongoing randomized trials such as the AIBL Active Trial [85] and the brain-in-motion trial [86] designed to address the relationship between physical exercise intervention and brain function in at risk individuals will help answer these questions.
